# Mutational studies on single circulating tumor cells isolated from the blood of inflammatory breast cancer patients

**DOI:** 10.1007/s10549-017-4176-x

**Published:** 2017-03-07

**Authors:** Catherine Bingham, Sandra V. Fernandez, Patricia Fittipaldi, Paul W. Dempsey, Karen J. Ruth, Massimo Cristofanilli, R. Katherine Alpaugh

**Affiliations:** 1 Fox Chase Cancer Center, Philadelphia, PA 19111 USA; 2 Cynvenio Biosystems, Westlake Village, CA USA; 3 Protocol Support Laboratory, Fox Chase Cancer Center, 333 Cottman Ave., Philadelphia, PA 19111 USA; 4 Robert H Lurie Comprehensive Cancer Center of Northwestern University, Chicago, IL USA

**Keywords:** CTC, Single-cell analysis, Tumor heterogeneity, IBC

## Abstract

**Purpose:**

The molecular characterization of circulating tumor cells (CTCs) is critical to identify the key drivers of cancer metastasis and devising therapeutic approaches, particularly for inflammatory breast cancer (IBC) which is usually diagnosed at advance stages and progresses rapidly.

**Methods:**

Genomic alterations in tumor tissue samples were studied using Foundation One™. Single CTCs were isolated using CellSearch followed by single-cell isolation by DEPArray™. Samples with 20 or more CTCs were chosen to isolate single CTCs using the DEPArray™.

**Results:**

Genomic alterations were studied in primary tumor or metastatic sites from 32 IBC patients. Genes with high-frequency mutations were as follows: TP53 (69%), RB1 (16%), PIK3CA (13%), and also ErbB2 (3%). At least once during treatment, CTCs were detected in 26 patients with metastatic IBC, in two patients with locally advanced IBC, and four patients had no detectable CTCs. Per 7.5 mL of blood, fifteen patients (47%) had ≥20 CTCs and six of them were chosen at random to isolate single CTCs. These cells were tested for the presence of TP53, RB1, PIK3CA, and/or ErbB2 mutations previously found in matching tissue biopsies. The isolated CTCs showed the same mutations as primary or metastatic tumor samples. Intra-patient CTC heterogeneity was found by the presence of different CTC subclones, with some CTCs harboring different combinations of mutated and wild-type genes.

**Conclusions:**

Our results indicate that CTCs could represent a non-invasive source of cancer cells from which to determine genetic markers as the disease progresses and identify potential therapeutic targets in IBC patients.

**Electronic supplementary material:**

The online version of this article (doi:10.1007/s10549-017-4176-x) contains supplementary material, which is available to authorized users.

## Introduction

Inflammatory breast cancer (IBC) is a very aggressive type of advanced breast cancer with a poor prognosis. The clinical symptoms of IBC involve the rapid onset of changes in the skin overlaying the breast, including edema, redness, and swelling, exhibiting a wrinkled, orange peel-like appearance of the skin known as peau d’orange [1]. This peculiar presentation is associated with the invasion of aggregates of tumor cells, defined as tumor emboli, into the dermal lymphatics, where they obstruct the lymph channels [2, 3]. IBC currently accounts for only 2–6% of all breast cancer cases in the United States and up to 20% of all breast cancer cases globally [4–7]. Due to its propensity to rapidly metastasize, it is responsible for a disproportionate number (15%) of breast cancer-related deaths [7–9]. IBC is either stage III or IV; at the time of diagnosis, virtually all patients have lymph node metastases and one third of the patients have metastases in distant organs such as the brain, the bones, and/or the visceral organs [6].

Metastatic disease is the most common cause of cancer-related death in patients with solid tumors and it is often associated with the presence of circulating tumor cells (CTCs) in the peripheral blood of cancer patients [10]. CTCs have been detected in a majority of epithelial cancers, including prostate [11], colorectal [12], and breast cancers [13]. CTCs are tumor cells shed from either the primary tumor or its metastases and can thus be regarded as “liquid biopsies” of metastasizing cells. Although their exact composition is unknown, a fraction of these cells is thought to be viable metastatic precursors capable of initiating a clonal metastatic lesion [14, 15]. Little is known about the timing of CTC release from primary tumors, their functional properties, or their heterogeneity. Intra-tumor heterogeneity denotes the coexistence of subpopulations of cancer cells that differ in their genetic, phenotypic, or behavioral characteristics within a given primary tumor and between a given primary tumor and its metastasis. Thus, intra-tumor heterogeneity poses a tremendous challenge for the characterization of biomarkers and treatments selection. In this work, we isolated single CTCs from the blood of IBC patients in order to analyze the presence of different mutations found in the primary tumor or metastatic sites and determine the heterogeneity of these cells.

## Materials and methods

### Patients

Thirty-two patients with inflammatory breast cancer (IBC) undergoing systemic treatment for their disease were included in this study. At the time of the first CTC enumeration, 29 patients had metastatic IBC (Stage IV) and three patients had locally advance IBC (Stage III). Clinical details and treatment timelines for the 32 patients are given in Supplementary Information. Targeted treatment outcomes have also been reported elsewhere on patients D84455 [16] and I77438 [17].

### Genomic studies in tumor samples

Formalin-fixed paraffin embedded (FFPE) tumor tissues (breast, chest wall, lymph node, bone marrow, liver biopsy, abdominal skin punch, brain biopsy, and/or pleural fluids) were used for genomic studies. Ten unstained sections were cut (5–10 µm) and placed on charged slides and submitted to Foundation Medicine (Cambridge, MA) for genetic analysis. Briefly, DNA was isolated from the fixed tumor cells and genomic analysis was performed using next-generation sequencing (NGS) (Foundation One™).

### CTCs enumeration from the blood using CellSearch

One tube of 7.5 mL blood from the IBC patients was drawn and the CellSearch™ System was used for CTC enrichment and enumeration. After running the blood in the CellSearch™ system for CTC enumeration, the cells were recovered from the cassettes in order to be used for single CTC selection using the DEPArray™ System (Silicon Biosystems, San Diego, CA). The standard protocol used for CTC enrichment is described in Supplemental Materials and Methods. Samples containing a minimum of 20 CTCs were selected and prepared for single-cell selection using the DEPArray™.

### Isolation of single CTCs using the DEPArray™ system

After the CellSearch enrichment, single CTCs were selected and isolated using the DEPArray™ (Silicon Biosystems) as described in Supplemental Materials and Methods. Individual CTCs or clustered cells that were α-cytokeratin (PE)-positive, CD45 (APC)-negative, and DAPI-positive were recovered in several tubes for genomic analysis. In addition, individual white blood cells (WBC) classified as CD45 (APC)-positive, CK (PE)-negative, and DAPI-positive were selected and recovered as single cells to use as controls in the genomic studies. Selected cells were stored at −80 °C for genomic analyses.

### TP53, ErbB2, PIK3CA, and RB1 mutations in CTCs

Whole genome amplification (WGA) was performed using the Ampli1™ WGA Kit (Silicon Biosystems). The Ampli1™ WGA kit uses a polymerase with proofreading activity with a lower error rate (4.8 × 10^−6^) than standard Taq DNA polymerases. Global amplification consisting of DNA isolation, restriction digestion, adaptor ligation, and PCR amplification was performed as described in Supplemental Materials and methods [18]. To study TP53, ErbB2, and PIK3CA mutations in CTCs, reverse and forward primers were used (Supplementary Table 1). The PCR products were cleaned using the QIAquick PCR purification kit and sequenced using the ABI 3130XL capillary genetic analyzer. As we were unsuccessful in studying RB1 K720* mutation using specific primers as described before for other genes, this mutation was studied using next-generation sequencing (NGS) as described in Supplemental Materials and Methods.

## Results

### Mutation analysis of tissue samples from metastatic IBC patients

A total of 32 patients with IBC were included in the study (Tables 1 and 2). All the patients were females save one male (B87480, Table 1), and all of them were at an advanced clinical stage at time of diagnosis (stage III or IV). Their median age at diagnosis was 48 years with a range of 32–72 years old. From the 32 patients, 20 patients (62.5%) had triple-negative (ER-negative, PgR-negative, and Her2-negative) IBC (Table 1); five patients (15.6%) had ER-positive Her2-negative IBC; three patients (9.4%) had ER-negative Her2-positive IBC; and four patients (12.5%) had ER-positive Her2-positive IBC at time of diagnosis (Table 2).

**Table 1:** Mutations in tumor samples and number of CTCs detected during disease progression in triple-negative IBC patients

Mutations and gene amplifications in tumor samples are shown. Time of tissue collection (*t* = 0; time of diagnosis) is indicated in each case. The survival time since IBC diagnostic is indicated. Several blood samples were run in order to determine the number of CTCs along the patient’s treatment; in the table, only the highest numbers of CTCs during each 10 month period are indicated (in Supplementary Information, all the points are shown). Also, the disease stage at the time of CTC enumeration is indicated

In some patients, CTCs were present as clusters; presence of CTCs clusters are indicated as (+). Baseline: CTCs number before neoadjuvant treatment; NDA no data available because the patient was treated at another institution; ND not-done

The time when patients came to Fox Chase Cancer Center for treatment recommendations and/or blood samples draw for CTC enumeration are indicated as (†)

**Table 2:** Mutations in tumor samples and number of CTCs detected during disease progression in ER-positive Her2-negative, ER-negative Her2-positive, and ER-positive Her2-positive IBC patients

Mutations and amplifications in tumor samples are shown. The age of the patients, the ER/PgR/Her2 status at diagnosis, and survival time since IBC diagnosis are indicated

The number of CTCs present in 7.5 mL of blood was determined using the CellSearch system

Blood samples were run in order to determine the number of CTCs during the disease progression; only the highest numbers of CTCs, every 10-month period since time of start of treatment, are showed

*NDA* no data available because the patient was treated in another institution, *ND* non-done, + presence of clusters

Baseline number of CTCs before treatment

The genomic alterations in the primary tumor or metastatic sites were determined using the NGS-based cancer gene test, Foundation One™ (Tables 1 and 2). IBC patients showed mutations in the following: TP53 (22/32; 69%), RB1 (5/32; 16%), PIK3CA (4/32; 13%), BRCA1 (3/32; 9%), BRCA2 (3/32; 9%), and Notch1 (3/32; 9%). Other mutated genes were ErbB2 (or Her2; 3%), ATM, Kras, ESR1, EGFR, and PAX5. Also IBC tumor samples showed amplifications in the following: MYC (8/32; 25%), CCND1 (7/32; 22%), ErbB2 (5/32; 16%), MCL1 (6/32; 19%), and FGFR1 (3/32; 9%). Interestingly, patient D66122, who was found to have a triple-negative disease by the analysis of her first biopsy, a second chest wall biopsy in month 26 showed amplification of the ErbB2 gene. Based on these results, this patient was subsequently treated with Herceptin (Table 1 and Supplementary Information).

### Circulating tumor cells (CTCs) enumeration

**Fig. 1:**
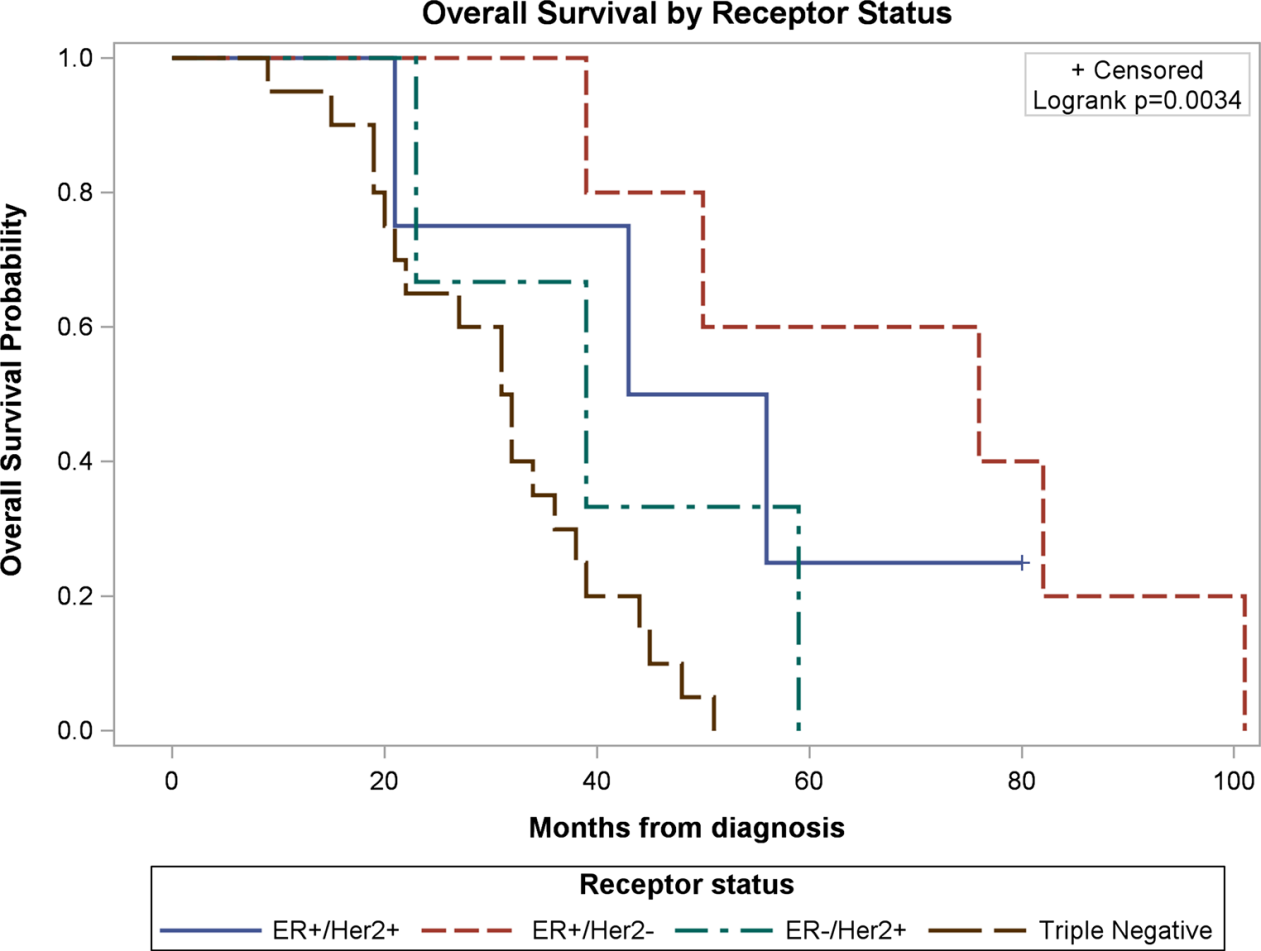
Survival curves according to ER (estrogen receptor) and Her2 (ErbB2) status. Overall survival in the triple-negative group was lower than the other groups (TN vs ER− Her2+, *p* = 0.011; TN vs ER+ Her2+, *p* = 0.001; TN vs ER+ Her2−, *p* = 0.027). Patients from the ER+ Her2− group had the longest median survival (76 months, 95% CI 39–101). In the ER+ Her2+ group, one patient was still alive and shown as censored (+) at the end of the curve

The number of CTCs present in 7.5 mL of blood was determined at different points during the patients’ treatments using the CellSearch™ system. From the 32 IBC patients, CTCs were detected in 28 patients (≥5 CTCs/7.5 mL blood in 24 patients; <5 CTCs/7.5 mL blood in four patients) at least once during treatment, while four patients had no detectable CTCs at any point during treatment (Tables 1 and 2). CTCs were detected in 26 patients with metastatic IBC and two patients with locally advance IBC. From 20 patients with triple-negative IBC, 16 patients had ≥5 CTCs/7.5 mL blood, and four patients had 0–4 CTCs/7.5 mL blood. Patients with triple-negative IBC had significantly worse overall survival compared to those with ER-positive Her2-positive IBC (*p* = 0.011), ER-positive Her2-negative IBC (*p* = 0.001), and ER-negative Her2-positive IBC (*p* = 0.027) (Fig. 1). Patients in the ER-positive Her2-negative group had the longest median survival (ER-positive Her2-negative = 76 months (95% CI 39–101), Triple-Negative = 31.5 months (95% CI 20–38), ER-negative Her2-positive = 39 months (95% CI 23–59.), ER-positive Her2-positive = 49.5 months (95% CI 21-undet.) (Figure 1). In the ER-positive Her2-positive group at the time of diagnosis (Table 2), patient K76386 developed a ER-positive (weak) Her2-negative component in month 9; and a tumor biopsy performed from patient B62630 in month 49 showed that the tumor was ER-positive Her2-negative (Supplementary Information). CTCs were detected in 26 patients with metastatic IBC (Stage IV) and in two patients with locally advance IBC (Stage III) at least once during treatments (Tables 1 and 2). CTCs were not detected in patient B87480 with triple-negative IBC (Table 1) nor in patients S71769, I77438, and J70105 with Her2-positive IBC (Table 2).

In order to successfully isolate single CTC using the DEPArray, samples from the CellSearch that contain at least 20 CTCs were used. Per 7.5 mL of blood from the 32 IBC patients, fifteen (47%) patients had ≥20 CTCs, two (6%) patients had 10–20 CTCs, five patients (16%) had between 5 and 10 CTC, and six patients (19%) had 1–5 CTCs. Of the 20 patients with triple-negative IBC, CTCs were detected in 19 of them at some point during the course of their disease, and 11 of these patients (55%) had more than 20 CTCs per 7.5 mL of blood (Table 1). CTCs were detected in all five patients with ER-positive Her2-negative IBC at least once during their treatment and two of these patients had more than 20 CTCs per 7.5 mL of blood (Table 2). Of the seven patients with Her2- positive IBC, three patients did not show any CTCs (although CTCs were enumerated only once in them), and two patients had more than 20 CTCs (Table 2). The two ER-positive Her2-positive IBC patients with a high number of CTCs were initially responsive to Herceptin therapy but during disease progression they failed to respond to the treatment (K76386 and B62630; Table 2).

### Mutations in single circulating tumor cell (CTC)

**Fig. 2:**
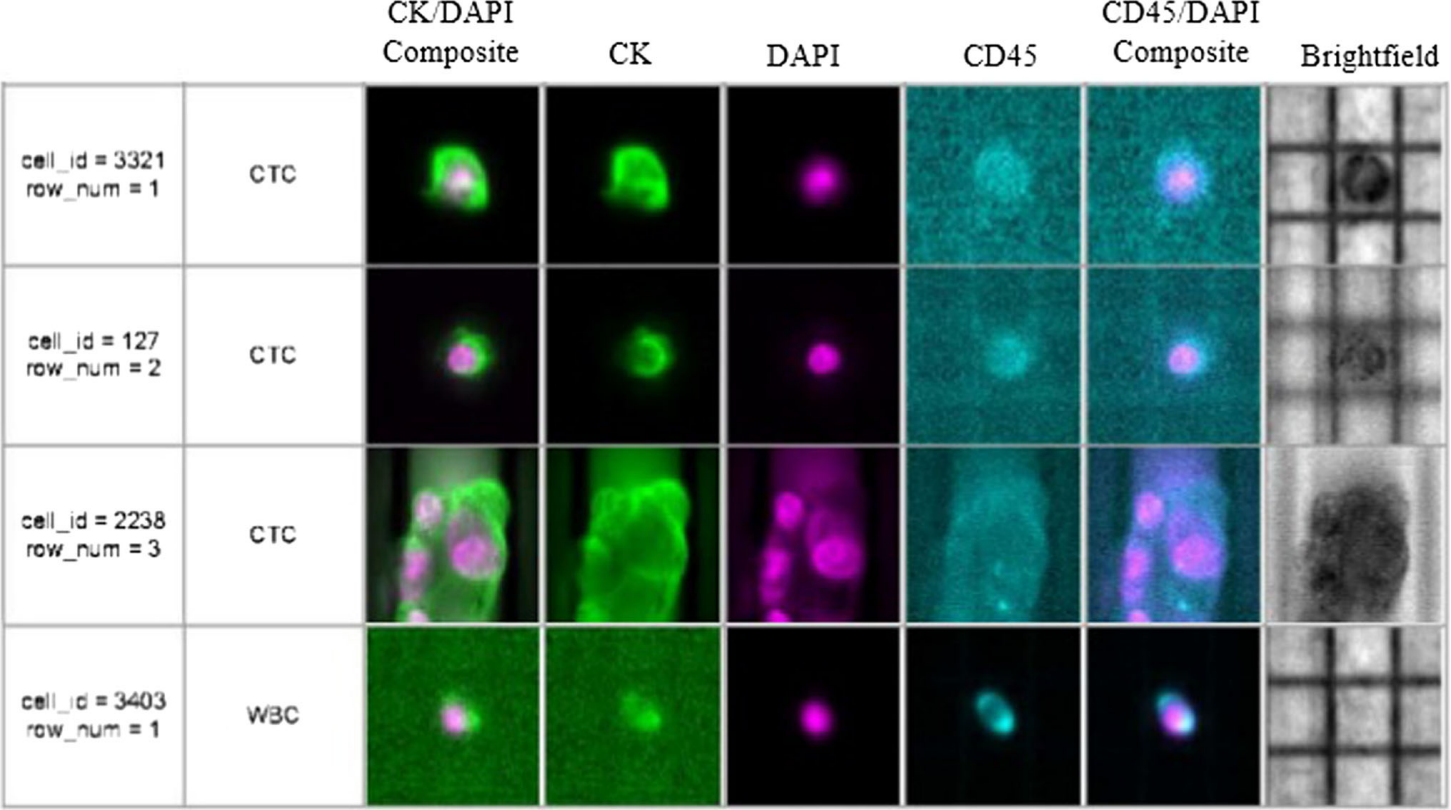
Single and clusters of CTCs from a patient with triple-negative metastatic IBC visualized in the DEPArray™. Tumor cells were defined as presence of a clear DAPI-stained nucleus, CK-PE-positive cytoplasm, and CD-45-APC negativity. Separate images for PE (*green*), DAPI (*magenta*), APC (*blue*) fluorescence, bright field channels, and merged CK-PE/DAPI and CD45-APC/DAPI images are shown. Single CTCs and a cluster of CTCs from a metastatic triple-negative IBC patient (D84455) are shown; also, four white blood cells (WBC) collected to use as controls for the molecular studies are shown

From the fifteen patients that had ≥20 CTCs, six were chosen at random in order to isolate single CTCs using the DEPArray™ (Table 3). Among these patients, all but patient B62630 had triple-negative IBC at diagnosis but, developed a Her2-negative component during disease progression as it was previously mentioned (Supplementary Information). For molecular characterization of single CTCs, cells were recovered from the CellSearch cassettes after enumeration as it was described in Supplementary Materials and Methods, washed and loaded in the DEPArray cassettes for single-cell isolation. The DEPArray™ system (Silicon Biosystems, San Diego, CA) is an automated platform that uses dielectrophoresis and a high-quality image-based cell selection system that allows for the identification and recovery of individual cells from heterogeneous samples [18]. CTCs that were discrete single cells were seen in the blood of three patients (J73299, R85453 and T77549), whereas the remaining three patients (B62630, L67504, and D84455) had both individual single CTCs and clusters of associated CTCs (Table 3). Usually, CTCs clusters were composed by five to 14 associated cells (Fig. 2). Samples containing single CTCs, pooled single CTCs, and/or CTCs clusters were selected and recovered using DEPArray™, and the samples were tested for the presence of mutations previously revealed in tumor samples from the same patient. WGA was performed on the isolated CTCs and regions of TP53, ErbB2, and PIK3CA shown to be mutated in matching tumor tissue were amplified and sequenced using Sanger’s method. In five of the six IBC patients where TP53 was mutated in the tumor tissues, the isolated CTCs showed the same TP53 mutations (Table 3). However, a TP53 S215G mutation was present in a chest wall biopsy in patient B62630, which was not observed in either the isolated CTCs or in tumor cells collected from pleural effusion (Table 2, pleural effusion from month 50). The TP53 S215G mutation is a missense mutation in exon 6 (Table 3). The other patients, J73299, R85453, L67504, and D84455, had TP53 mutations that produced a premature stop codon in the protein (Table 3). The liver biopsy of patient J73299 showed a mutation in TP53 exon 6, TP53 P190_H193 > *E; this mutation was also found in CTCs isolated from the patient’s blood (Table 3). Two patients (R85453 and L67504) had a TP53 mutation in exon 4, TP53 R110 fs*13 (Table 3). In patient L67504, a C deletion (nucleotide 328, delC) was detected in the chest wall biopsy and the same mutation was found in the CTCs (Table 3). Patient R85453 showed a G deletion (nucleotide 329, delG) in p53 exon 4 in a breast biopsy sample (Table 1); while this mutation was also found in one CTC isolated from the patient, a second CTC revealed a C deletion (nucleotide 328, delC) that was not previously detected in the tissue biopsy (Table 3). The chest wall biopsy of patient D84455 showed a TP53 C229 fs*10 mutation that was also found in three of five single CTCs analyzed and in one CTC cluster isolated from the patient’s blood; one single CTC showed the TP53 wild-type allele and one CTC provided no data as we were unable to successfully amplify the region of interest (Table 3). In Table 4, the deleterious mutations in TP53 are shown.

**Table 3:** Mutation analyses in single, pooled, and/or CTCs clusters

Single CTCs, pooled single CTCs, and/or CTCs clusters were recovered using DEPArray. Mutations in TP53, ErbB2, PIK3CA, and RB1 found in tumor tissue samples were detected in the CTCs

Intra-patient heterogeneous CTCs populations were found; w.t. wild type; n.d. non-done (because not enough amplified DNA)

**Table 4:** TP53 deleterious mutations in IBC tumor and CTCs

The TP53 P190_H193>*E mutation is a 12 base pair (bp) deletion that causes the loss of amino acids at position 190 (P) to 193 (H), and the insertion of 6 nucleotides (TAA GAG) that produce a premature stop codon in the protein

TP53 R110 fs*13 is a frame shift deleterious mutation that causes a stop codon at position 122 (nucleotide 366) of TP53 with the loss of the protein

The TP53 C229 fs*10 is a mutation in exon 7 of TP53 consisting in a 2 bp. deletion

The RB1 gene was mutated at high frequency in IBC patients; CTCs isolated from patients T77549 and L67504 were chosen to further study RB1 mutations in single CTCs (Table 3). RB1 splice mutation 607 + 1 G > C was detected in the chest wall biopsy from T77549 and this mutation was detected in one single CTC and in one pool of 6 CTCs isolated from the blood of that patient (Table 3). A second pooled cell sample containing 4 CTCs harbored RB1 wild type. The RB1 607 + 1 G > C denotes the G to C substitution at nucleotide +1 of the intron (between exons 6 and 7) in the coding DNA positioned between nucleotide 607 and 608, resulting in low expression or partial inactivation of the RB1 protein. Patient L67504 showed a RB1 K720* mutation in her chest wall (Table 1); the RB1 K720* mutation was also found in two of five single CTCs and a cluster containing three CTCs that were isolated from the blood (Table 3). The RB1 K720* mutation is a nonsense variant, a substitution that produces an immediate stop codon. This patient also showed a BRCA2 p.A1326 fs*4 mutation in her chest wall biopsy (Table 1); the BRCA2 p.A1326 fs*4 mutation was detected in one of three white blood cells (WBC) used as controls, suggesting that the BRCA2 mutation is a germline mutation. This mutation was not investigated in the isolated CTCs. In both patients that had RB1 mutations, some CTCs showed the RB1 mutations and others showed the wild-type RB1 allele (Table 3).

The chest wall biopsy from patient D84455 had two mutations in ErbB2: S310F in exon 12, and V777L in exon 24; this patient also had a mutation in PIK3CA K111E, in addition to the TP53 C229 fs*10 mutation previously described (Table 3). These mutations were studied in five single CTCs and one CTC cluster. Two single CTCs and the cluster showed all the mutations detected in the chest wall biopsy; another single CTC only showed the ErbB2 mutations (ErbB2 S310F and ErbB2 V777L) and wild-type alleles of TP53 and PIK3CA. Another single CTC showed the ErbB2 mutation on exon 12 (ErbB2 S310F), the wild-type allele on exon 24, but the amplified DNA was not enough to study TP53 and PIK3CA in this cell (Table 3). The remaining single CTC showed the mutated TP53 (TP53 C229 fs*10) and ErbB2 exon 24 wild type. Results indicated that patients D84455 and L67504, in which mutations in more than one gene were studied, had a heterogeneous population of CTCs (Table 3).

## Discussion

Genomic alterations were studied in primary and/or metastatic tumor samples from 32 patients with IBC. Most of the patients had triple-negative IBC and had mutations in TP53, RB1, and/or PIK3CA. CTCs were detected in 28 of the 32 IBC patients included in this study. Single CTCs isolated from the blood of six of these patients showed the same mutations as primary or metastatic tumor samples indicating that CTCs can potentially be used to monitor disease progression. Our results show that mutations in driver genes found in the primary tumor and/or the metastasis in IBC patients could be identified in the CTCs. Furthermore, the mutational analysis of the TP53, RB1, PIK3CA, and ErbB2 genes revealed heterogeneity of CTCs.

Deleterious TP53 mutations found in tissue samples or pleural effusions of IBC patients were also present in CTCs isolated from their blood. In the majority of the IBC patients with TP53 mutations, these mutations resulted in a non-functional protein. A non-functional TP53 has been shown to offer survival advantages to the cancer cells by facilitating growth, anoikis resistance, and the emergence of a potentially more aggressive malignancy [19]. TP53 mutations are exceptionally frequent in cancer and are among the key driving factors in triple-negative breast cancer (TNBC) [20]. Furthermore, TP53 mutations are more frequent in inflammatory breast cancer (50%) than in non-inflammatory breast cancer (20–30%) [21, 22]. TP53 mutations have been shown to predict a poor response to anthracycline-based neoadjuvant chemotherapy [23–25]; others suggested that TP53 mutations confer sensitivity to taxane [26, 27]. A recent study suggested that patients with TP53 mutations are more likely to respond to anthracycline/cyclophosphamide-based neoadjuvant chemotherapy [28]. Several clinical trials are ongoing to study TP53-mutated breast cancer sensitivity to different chemotherapeutic agents. Other clinical trials are targeted towards either expression of the wild-type TP53, suppressing expression of mutated TP53, or strategies that involve targeting of the cell cycle regulator Wee-1 tyrosine kinase inhibitors (clinicaltrials.gov).

RB1 mutations were found in only the triple-negative IBC patients from this study, and all of these mutations render a premature stop codon and a non-functional RB1 protein. RB1 mutations were also detected in the CTCs. RB1 mediates cell cycle control and is frequently inactivated in human TNBC [29–31]. There are targeted inhibitors that are currently in advanced clinical testing for tumors harboring RB1 and PIK3CA mutations [32, 33].

One triple-negative IBC patient (D84455) with a deleterious TP53 mutation also showed two ErbB2 mutations (V777L and S310F). Both mutations activate ErbB2 by either affecting its auto-phosphorylation or phosphorylation of downstream substrates in breast cancer cells [34–36]. Initially, this patient had ER+ Her2+ invasive ductal carcinoma (IDC) in the left breast that was treated with standard local therapies over 2 years and this patient subsequently developed triple-negative IBC in the same breast [16] (Supplementary Information). This suggests that the ErbB2 pathway could be the driver of this patient’s disease even in the absence of ErbB2 amplification. Furthermore, this patient had a PIK3CA K111E mutation which is also an activating mutation [37, 38]. All the mutations detected in the chest wall biopsy of patient D84455 were detected in the CTCs isolated from their blood. A heterogeneous population of CTCs were found in this patient with some CTCs showing the mutated genes and others showing different combinations of the mutated and wild-type genes.

It has been successfully demonstrated by single-cell sequencing that many breast cancers are composed of multiple distinct subclones [39]. Intra-patient cellular heterogeneity is widely reported in epithelial malignancies and it is expected that CTCs will also be heterogeneous [40–42]. Our results are consistent with previous findings which showed a heterogeneous pattern of genomic mutations on single CTCs obtained from breast, esophageal, and colorectal cancer patients [42–44].

Our results demonstrate that we were able to select uncontaminated CTCs by combining the CellSearch and DEPArray™ systems. However, one disadvantage of the DEPArray™ is that there is approximately 40% cell-loss, although the DEPArray cartridge is manually loaded with 14 μL of sample, only 9.26 μL of sample is injected into the micro-chamber of the cartridge. We found that in order to successfully isolate single CTC, samples with 20 CTCs or more should be used to load in the DEPArray cassette. In order to perform molecular analysis of CTCs in samples with less than 20 CTCs per 7.5 mL blood, multiple samples from the same patient could potentially be combined after the CellSearch and loaded in the DEPArray cassette.

However, despite the promise of CTCs as multifunctional biomarkers, there are still numerous challenges that hinder their incorporation into standard clinical practice. Some patients showed little to no CTCs even though their disease was progressing. This is the case for most of the patients with Her2-positive IBC, with the exception of patients K76386 and B62630 who developed a Her2-negative component during their disease progression; both had high number of CTCs. Patients with Her2-positive IBC were treated with Herceptin for a long period of time, so this could explain why CTCs were not present in their blood due to the high specificity of these antibodies. In addition, the CellSearch™, relies on the detection of the surface epithelial cell adhesion molecule EpCAM, and the existence of an EpCAM-negative subpopulation of CTCs had been described in patients with Her2-positive metastatic breast cancer [45]; therefore, it will be interesting to combine different pre-enrichment strategies with the DEPArray in order to study these cells, especially in Her2-positive IBC.

It has been shown that elevated CTC at baseline or at any time through the course of metastatic breast cancer is associated with worse prognosis; patients with ≥5 CTCs/7.5 mL blood had a shorter overall survival compared with the patients with <5CTCs/7.5 mL blood, and elevated CTCs while on treatment ultimately are predictive of an ineffective therapy [13, 46]. Our data showed that the distribution of CTCs in the patients with serial blood draws during the treatments varied; many patients were initially CTCs negative but converted to positive and vice versa. Based on our results, single time-point measurements of CTCs seem to be inadequate, and could result in incorrect microscopic disease staging. Collecting sequential blood samples for real-time monitoring of the efficacy of systemic therapies would offer new possibilities in evaluating targeted therapies based on genomic profiling of CTCs and improving the clinical management of patients with IBC. In order to implement these studies in future clinical practice, we are developing protocols in order to study mutations in single, pools, and clusters of CTCs using NGS and a panel of 15 genes frequently mutated in IBC.

This work demonstrates that the isolation and pooling of CTCs from IBC patients can be used for genomic analysis, both to initially identify targetable mutations where solid tumor samples are unavailable and to be used as a biomarker to reveal which cell populations are affected by the current or previous therapy. Our results suggest that CTCs represent the entire spectrum of the primary tumor and distal metastases for patients with IBC. Furthermore, our studies showed the presence of different CTCs subclones in the peripheral blood of IBC patients.

## Electronic supplementary material

Below is the link to the electronic supplementary material.

Supplementary material 1 (DOCX 13 kb)

Supplementary material 2 (DOCX 16 kb)

Supplementary material 3 (DOCX 80 kb)

## Abbreviations

CK: Cytokeratin
CTC: Circulating tumor cell
EpCAM: Epithelial cell adhesion molecule
ErbB2 or Her2: v-erb-b2 avian erythroblastic leukemia viral oncogene homolog 2
FFPE: Formalin- fixed paraffin embedded
Fs: Frameshift
IBC: Inflammatory breast cancer
NGS: Next-generation sequencing
OS: Overall survival
PE: Phycoerythrin
PgR: Progesterone receptor
RB1: Retinoblastoma tumor suppressor
TN: Triple-negative
TNBC: Triple-negative breast cancer
WBC: White blood cells
WGA: Whole genome amplification
