# Supplementary material for: Mutational studies on single circulating tumor cells isolated from the blood of inflammatory breast cancer patients
Source: Breast Cancer Res Treat. 2017 Mar 7;163(2):219–30. doi: 10.1007/s10549-017-4176-x (PMC5410214; doi:10.1007/s10549-017-4176-x)
Supplement: Supplementary file 1 — Supplementary material 1 (DOCX 13 kb) [file 10549_2017_4176_MOESM1_ESM.docx]

| ***Gene*** | ***Location*** | ***Aminoacid*** | ***Primer reverse*** | ***Primer forward*** | ***PCR product*** |
| --- | --- | --- | --- | --- | --- |
| ***TP53*** | ***Exon 4*** | ***S99*** | ***5’ AGGCATTGAAGTCTCATGGAAG 3’*** | ***5’CAATGGATGATTTGATGCTGTC 3’*** | ***304 bp*** |
|  |  |  | ***5’ AGGCATTGAAGTCTCATGGAAG 3’*** | ***5’ GTTCTGGTAAGGACAAGGGTTG 3’*** | ***438 bp*** |
| ***TP53*** | ***Exon 4*** | ***R110*** | ***5’ AGGCATTGAAGTCTCATGGAAG 3’*** | ***5’CAATGGATGATTTGATGCTGTC 3’*** | ***304 bp*** |
|  |  |  | ***5’ AGGCATTGAAGTCTCATGGAAG 3’*** | ***5’ CCTTCCCAGAAAACCTACCAG 3’*** | ***128 bp*** |
| ***TP53*** | ***Exon 6*** | ***P190*** | ***5’ CAGTTGCAAACCAGACCTCAG 3’*** | ***5’ CCGTCTTCCAGTTGCTTTATCT 3’*** | ***455 bp*** |
| ***TP53*** | ***Exon 6*** | ***S215*** | ***5’ CAGTTGCAAACCAGACCTCAG 3’*** | ***5’ CCGTCTTCCAGTTGCTTTATCT 3’*** | ***455 bp*** |
| ***TP53*** | ***Exon 7*** | ***C229*** | ***5’ ACTGAGTGGGAGCAGTAAGGAG 3’*** | ***5’CTCATCTTGGGCCTGTGTTATCT 3’*** | ***298 bp*** |
| ***RB1 607+1***  ***(codon 202)*** | ***Exon 6***  ***(Splice)*** | ***-----*** | ***5’ ATTCCTGCTGTCAGTTAATAAATATAATGAAC 3'*** | ***5'GTCAGTTAATAGGATATCTACTGAAATAAATTCTG 3'*** | *93 bp* |
| ***ErbB2*** | ***Exon 12*** | ***S310F*** | ***5’ TGTGTTCCATCCTCTGCTGTC 3’*** | ***5’ GTAATGCTGCTCATGGTGGTG3’*** | *151 bp* |
| ***ErbB2*** | ***Exon 24*** | ***V777L*** | ***5’ ATGGTCTAAGAGGCAGCCATAG 3’*** | ***5’ GTGTGGTCTCCCATACCCTCTC 3’*** | *161 bp* |
| ***PIK3CA*** | ***Exon 2*** | ***K111E*** | ***5’ GCTTTATGGTTATTTGCATTTTAGA 3’*** | ***5’ ACCAGTAGGCAACCGTGAAG 3’*** | ***89 bp*** |

**Table S1. Primers used to amplified TP53, RB1, ErbB2 and PIK3CA.**
