# Supplementary material for: Mutational studies on single circulating tumor cells isolated from the blood of inflammatory breast cancer patients
Source: Breast Cancer Res Treat. 2017 Mar 7;163(2):219–30. doi: 10.1007/s10549-017-4176-x (PMC5410214; doi:10.1007/s10549-017-4176-x)
Supplement: Supplementary file 2 — Supplementary material 2 (DOCX 16 kb) [file 10549_2017_4176_MOESM2_ESM.docx]

**Supplemental Material and Methods**

**Patients**

Patients included in this study came to Fox Chase Cancer Center during the period from August 2010 through December 2012.

**Statistical analysis**

For each receptor group, overall survival curves were generated using Kaplan Meier methods from date of diagnosis. Overall survival was compared in the four groups using the log rank test; pair wise comparisons with the Triple Negative receptor group were adjusted using Bonferroni correction for multiple comparisons. CTC counts by liver metastases status were compared using a Kruskal-Wallis non-parametric test. Statistical analyses were performed using SAS/STAT software, version 9.4.

**Genomic studies in tumor samples**

Foundation One^TM^ is a comprehensive, NGS-based, cancer gene test which is routinely applied to FFPE clinical samples. The assay sequences the entire coding sequence of 182 cancer-related genes (3,230 exons) and 37 introns from 14 genes frequently rearranged in cancer; gene mutations and amplifications were determined. 50 ng of DNA extracted from FFPE tumor specimens and sequenced to a unique median depth of >800x. Reads were mapped to human genome reference and alignment results were analyzed by customized tools.

**CTCs enumeration from the blood using CellSearch**

The CellSearch^TM^ System was used for CTC enrichment and enumeration. Briefly, CTCs were enriched using ferrofluid conjugated with anti-epithelial cell adhesion molecule (EpCAM) antibodies. Captured cells were stained with fluorescently labeled monoclonal antibodies specific for pan cytokeratin (CK-8/18/19-PE), leukocyte common antigen (CD45-APC), and nuclear stained with [4’, 6 diamidino 2 phenylindole (DAPI)] [18]. Tumor cells were defined using standard CellSearch CTC criteria: round to oval shape, presence of a clear DAPI-stained nucleus, at least 50% overlap between the CK-PE-positive cytoplasm and the nucleus and CD-45-APC negative.

**Sample transfer from CellSearch to DEPArray™ cartridges**

Cells were removed from the cartridges and transferred into a Protein LoBind tube (Eppendorf) and the cartridge was washed twice with elution buffer and all samples combined. The samples were centrifuged at 600 × g for 5 minutes and the supernatant was carefully removed. The cell pellets were stored and protected from light at 4°C in a residual volume of elution buffer until use. This procedure was performed on the same day the sample was run on the CellTracks, as delaying removal of cells from the cartridge decreased the number of CTCs recovered.

**Isolation of single CTCs using the DEPArrayTM system**

After the CellSearch enrichment, the CTCs were selected and isolated using the DEPArray™ (Silicon Biosystems). The system is an automated platform that uses dielectrophoresis and a high-quality image-based cell selection system that allows for the identification and recovery of individual cells from heterogeneous samples. The DEPArray™ chip consists of various microelectrodes that create electric cages into which individual cells are trapped and, by alternatively activating and deactivating the microelectrodes, the cells are moved to a position in the chip that allows their recovery. Briefly, DEPArray™ cartridges (DEPArray™ A300K-cartridge, Silicon Biosystems) were loaded with 800 μl of SB115 buffer and 14 μl of sample, placing approximtely 9.26 μl of sample within the electrophoretic chamber. Upon the application of a preprogrammed electric field, cells moved to and were held within their nearest electrically controlled cages. The cartridges allowed for either 16,000 or 40,000 cells to be trapped. Images of each cage were captured with white light exposure and each of three fluorescent filter cubes (PE, APC, and DAPI/Hoechst). Cells were automatically detected by the system based on a DAPI/ Hoechst fluorescence threshold and assigned a unique cell ID. Captured images were digitally processed using multiple parameters outlined by the operator and presented in a software module that enables selection of cells of interest.

Next, in the recovery step, selected cells were electrically moved to a parking area adjacent to the main microchamber in the cartridge. Finally, individual cells were moved from the parking area for recovery and flushed from the chamber with three drops of SB115 buffer (30 to 40 μl) into a 200-μl PCR tube. The entire cell-routing process was monitored under bright field imaging. Routing and paths were automatically calculated by the software and routing parameters, such as speed, can be manually adjusted by the operator. A cell pellet was prepared as recommended by the supplier. Briefly, the tubes were spun at 14,000 × g for 10 minutes, 100 μl PBS was added and the sample was centrifuged again for 25 minutes. The buffer was removed from the tube and the cell pellets were stored at −80°C until further use for whole genome amplification (WGA). Individual CTCs or clusters, classified as α-cytokeratin (PE)-positive, CD45 (APC)-negative and DAPI-positive cells, were recovered in several tubes for genomic analysis. Also, individual white blood cells (WBCs) classified as CD45 (APC)-positive, CK (PE)-negative, and DAPI-positive cells, were selected and recovered as single cells to use as controls in the genomic studies.

**Whole genome amplification (WGA)**

To allow genotyping analysis of single CTCs, WGA was performed using the Ampli1™ WGA Kit (Silicon Biosystems). The Ampli1™ WGA kit uses a polymerase with proofreading activity with a lower error rate (4.8 × 10−6) with respect to standard Taq DNA polymerases. The isolated CTCs were thawed on ice and brought up to a volume of 1 μl for the WGA procedure; single WBCs were also subjected to WGA in order to use as controls for mutation analyses. Global amplification consisting of DNA isolation, restriction digestion, adaptor ligation and PCR amplification were performed as recommended by the supplier. Briefly, cells were lysed overnight and then digested with *MseI* restriction enzyme; adapters were ligated onto the digested DNA and fragments were then amplified by PCR with time and temperature gradients using adapter specific primers; the final volume of the sample after amplification was 50 μl. As control for the WGA, products were subjected to an end-point PCR for two control genomic DNA sequences of 373 and 167 bp, respectively (Ampli1™ QC kit; SB); 2 μl of the Amli1™ WGA product was used as template per reaction and PCR products were analyzed by gel electrophoresis on the Agilent 2100 Bioanalyzer using the DNA 1000 kit (Agilent Technologies, Santa Clara, CA, USA). Only samples that showed both bands were used to study mutations.

**TP53, ErbB2, PIK3CA, and RB1 mutations in CTCs**

To study TP53, ErbB2, and PIK3CA mutations in CTCs, primers reverse and forward were used (Supplementary Table 1). PCR reactions were performed using 2 µl of the Amli1^TM^ WGA product, GoTaq (Promega), 2 µM primers, and with an annealing temperature of 52°C. The PCR products were cleaned using the QIAquick PCR purification kit, and sequenced using the ABI 3130XL capillary genetic analyzer. The Sequencher software was used for sequences analyses. As we were unsuccessful to study RB1 mutations using specific primers as described before for other genes, RB1 mutations were studied using next generation sequencing (NGS). Briefly, the WGA product from CTCs or WBC were used to prepare the DNA library for NextGen Sequencing. The Ion AmpliSeq Cancer Hot Spot Panel version 2 panel, which targets about 2,800 specific mutations in 50 oncogenes and tumor suppressor genes, was used. The Ion Torrent PGM System (Thermo Fisher) was used and assembly and mutational analysis were completed using the DNASTAR software package.
