# Supplementary material for: Mutational studies on single circulating tumor cells isolated from the blood of inflammatory breast cancer patients
Source: Breast Cancer Res Treat. 2017 Mar 7;163(2):219–30. doi: 10.1007/s10549-017-4176-x (PMC5410214; doi:10.1007/s10549-017-4176-x)
Supplement: Supplementary file 3 — Supplementary material 3 (DOCX 80 kb) [file 10549_2017_4176_MOESM3_ESM.docx]

**Supplementary Information**

**Supplementary Data 1. Patient clinical histories.** The treatments and number of CTCs/7.5 mL of blood are indicated.

**Patient J73299**

Month 0: IBC diagnosed in the right breast; stage IIIB; T4d N1 M0; triple negative/ Month 1-4: neoadjuvant chemotherapy Adriamycin (doxorubicin) and Cytoxan (cyclophosphamide) every two weeks for four cycles followed by Taxol (paclitaxel) weekly for 12 weeks; stopped due to disease progression/ Month 4-7: neoadjuvant therapy carboplatin and gemcitabine for five cycles/ Month 5: patient presented to Fox Chase Cancer Center for treatment recommendations/ Month 8: right modified radical mastectomy and axillary lymph node dissection and left simple mastectomy; 0/10 positive lymph nodes; residual disease in the skin of the chest wall / Month 8-10: radiation therapy/ Month 11: no clinical evidence of recurrent disease/ Month 15: PET/CT scan showed recurrent disease in the abdomen, metastases in liver and abdominal lymph nodes; CTCs= 3 / Month 15-17: Ixempra (ixabepilone) and Xeloda (capacitabine)/ Month 17: stopped Ixempra and Xeloda due to disease progression in the liver; CTCs= 55/ Month 17-19: cisplatin and vinorelbine for three cycles; CTCs= 101* (month 18) / Month 19: PET/CT scan showed progression of the disease; new lesions in the liver; CTCs=44 (month 19)/ Month 19-20: cyclophosphamide and methotrexate, with lapatinib and metformin/ Month 20: developing ascites but not malignant cells were found / Month 21: CTCs= 201*; patient died.

*CTC values included in Table 1

**Patient T77549**

The patient had invasive ductal carcinoma (IDC) triple negative in the right breast two years before IBC diagnosis; 5/11 positive lymph nodes.

Month 0: IBC diagnosed in right breast; stage IIIC; triple negative/ Month 1: bilateral simple mastectomy; no evidence of carcinoma in the left breast / Month 2-9: adjuvant chemotherapy Taxotere (docetaxel) and carboplatin for eight cycles/ Month 10: patient developed chest wall recurrence and started Xeloda (capacitabine)/ Month 11: PET/CT scan showed increased activity in small left axillary and internal mammary lymph nodes; increased size of left axillary lymph node / Month 12: patient presented to Fox Chase Cancer Center for treatment recommendations; erythema extending from chest wall to the upper abdomen skin (Stage IV); started Ixempra (ixabepilone) every three weeks /Month 13: Ixempra; stopped due to increase activity on the lung; CTCs= 9/ Month 15: started epirubicin every three weeks; CTCs= 15/ Month 17: epirubicin stopped due to disease progression; erythema on her chest wall continued to extend; there was some progression in the activity of the left axilla and left upper chest wall, and the erythema on the left chest wall continued to increase; CTCs=3/ Month 18: started Phase 1 clinical trial AZD1480 Jak2 inhibitor; shortly after stopped due to disease progression; CTCs= 60* (month 18)/ Month 18-19: started Doxil (doxorubicin) and Cytoxan (cyclophosphamide)/ Month 20: PET/CT scan detected brain metastases and increased activity in lymph nodes and chest wall; CTCs= 49/ Month 20-21: started Abraxane (paclitaxel) and gemcitabine/ Month 21: CTCs= 85/ Month 22: PET/CT scan showed bone, liver, brain metastases; stopped Abraxane and gemcitabine; CTCs= 112/ Month 22-24: began cisplatin and Tykerb (lapatinib); stopped due to disease progression clinically/ Month 23: CTCs=347/Month 24-25: Metformin; stopped due disease progression; CTCs= 2,502*(month 24); CTCs= 2,226 (month 25)/ Month 25: patient died.

*CTC values included in Table 1

**Patient D84455**

The patient had invasive ductal carcinoma ER+ PR+ Her2+ of the left breast two years before IBC diagnosis. She received Femara (letrozole) which it was later switched to Aromasin (exemestane).

Month 0: IBC diagnosed in the left breast with lymph nodes (left neck, axilla, and mediastinal lymph nodes and a cervical lymph node) and skin involvement (Stage IV), triple negative/ Month 1-8: Taxol (paclitaxel) and Avastin (bevacizumab) / Month 8: Taxol dropped on month 8 due to neuropathy; continued on Avastin alone/ Month 11: Avastin stopped due to disease progression/ Month 12-15: started desatinib and Ixempra (ixabepilone); stopped due to disease progression (new skin involvement)/ Month 16: started gemcitabine and Taxotere (docetaxel)/ Month 17: patient presented to Fox Chase Cancer Center for treatment recommendations; stopped gemcitabine and Taxotere due to skin disease progression; CTCs= 25* (month 17)/ Month 17-20: started 5-FU, epirubicin, cyclophosphamide (FEC); CTCs= 3 (month 18)/ Month 20: FEC stopped due to skin disease progression; CTCs= 17 (CTCs in clusters)/ Month 20-23: started on carboplatin every three weeks; CTCs= 40 (month 21; CTCs in clusters); CTCs= 26 (month 23); stopped due to disease progression on month 23/ Month 24-28: started Herceptin (trastuzumab), Tykerb (lapatinib) and Abraxane (paclitaxel); CTCs=46* (month 24); stopped due to disease progression/ Month 31: CTCs= 19* (CTCs in clusters); patient died (see Ref. 16: Ali et al., J Clin Oncol 2014: 32, 88-91).

*CTC values included in Table 1

**Patient R85453**

Month 0: IBC diagnosed in the right breast; stage IIIB; T4d N1 M0; triple negative/ Month 1-7: dose-dense Adriamycin (doxorubicin) and cyclophosphamide followed by weekly Taxol (paclitaxel) for 12 weeks; MRI showed extensive persistent disease after chemotherapy/ Month 8: bilateral mastectomy; 15/20 positive lymph nodes/ patient had contralateral (left) carcinoma with 7/16 positive lymph nodes; PET/CT scan identified a hypermetabolic lymph node in the right supraclavicular / Month 9: patient presented to Fox Chase Cancer Center for treatment recommendations; CTCs= 33* (month 9)/ Month 9-12: started gemcitabine and carboplatin; stopped at month 12 due to poor response; CTCs=3 (month 11); CTCs=10 (month 12)/ Month 13: started Ixempra (ixabepilone) and Xeloda (capacitabine)/ Month 14: PET/CT scan showed multiple active lymph nodes, chest wall lesions, metastases in lungs, liver and bones; CTCs= 222* (month 14)/ Month 15: patient died.

*CTC values included in Table 1

**Patient L67504**

Month 0: IBC diagnosed in the left breast; stage IIIC; T4d N1 M0; triple negative/ Month 5-8: neoadjuvant chemotherapy with Adriamycin (doxorubicin) and carboplatin followed by Taxol (paclitaxel)/ Month 9: left modified radical mastectomy; 0/4 positive lymph nodes/ Month 10-12: radiation therapy completed/ Month 20: patient developed a left chest wall disease; shoulder recurrence (small area of rash) that was surgically removed and was compatible with metastatic disease; no additional therapy/ Month 21: patient developed an extensive skin rash on the same side chest wall and, biopsy proven to be recurrent IBC with extensive lymphovascular invasion; triple negative / Month 22: patient presented to Fox Chase Cancer Center for treatment recommendations; disease in the contralateral axillary lymph node as well as diffuse chest wall disease; no evidence of visceral disease; IBC Stage IV; CTCs= 20* (month 22)/ Month 22-28: started Ixempra (ixabepilone) and Xeloda (capecitabine); CTCs= 12 (month 23); CTCs= 0 (month 24); on month 26, good response to treatment with no evidence of metabolically active malignancy on two subsequent PET/CT scans; CTCs = 0 (month 26)/ Month 28: developed rash on left chest wall and left upper arm and was re-evaluated at FCCC; CTCs= 5 (month 28); skin biopsy of the left upper arm and clavicular areas consistent with metastatic carcinoma in dermal lymphatics / Month 30-31: CTCs= 9 (month 30); started phase I clinical trial JAK2 inhibitor AZD 1480; stopped AZD 1480 due to clinical disease progression; CTCs= 12* (month 31)/ Month 31- 33: started gemcitabine and carboplatin; stopped due to bone marrow toxicity; CTCs= 0 (month 33)/ Month 34-38: started Cytoxan (cyclophosphamide) and methotrexate; CTC=0 (month 35); stopped due to progression of the disease in the chest wall; CTCs=11 (month 38)/ Month 39-46: started Abraxane (paclitaxel); stopped due to chest wall disease progression; CTC= 0 (month 41); CTC= 8 (month 44); CTC= 45 (month 46)/ Month 46-47: started Taxotere (docetaxel); CTCs= 90* (month 47); PET-CT scan showed progression of disease with multiple new lesions in head/neck lymph nodes bilaterally,

as well as left iliac and inguinal lymph nodes; in addition, active lung lesions in the right upper lobe; started with cisplatin and Taxol (paclitaxel)/ Month 51: metastases in lung; no lesions in liver or bones; patient died.

*CTC values included in Table 1

**Patient T81354**

Month 0: IBC diagnosed in the right breast (T4d N1 Mx) and IDC (T2 N3c Mx) in the left breast, both triple negative/ Month 1: bone scan was negative for metastatic disease; a CT of the chest, abdomen and pelvis revealed widespread nodal metastases above the diaphragm; dose-dense Adriamycin (doxorubicin) and cyclophosphamide with Neulasta support for two cycles/ Month 2: patient presented to Fox Chase Cancer Center for treatment recommendations; Stage IV;CTCs= 9* (month 2)/ Month 2-7: started 5-fluorouracil, epirubicin and cyclophosphamide and completed 3 cycles; after Taxol (paclitaxel) weekly for 9 weeks; stopped due to disease progression in the lymph nodes and bones; CTCs=6 (month 7)/ Month 7-11: gemcitabine and carboplatin/ Month 11: bilateral mastectomy/ Month 12: complete clinical and functional response/ Month 15: completed bilateral radiation/ Month 16: CTCs=2 / Month 19: recurrent disease; CTCs= 4* (month 19)/ Month 20: patient went to another institution for a clinical trial with ARQ197 c-Met inhibitor/ Month 21-44: no data available/ Month 45: patient died.

*CTC values included in Table 1

**Patient S80274**

Month 0: IBC diagnosed in the right breast; stage IIIC; T4d N3 M0; triple negative/ Month 1-4: dose-dense Adriamycin (doxorubicin) and Cytoxan (cyclophosphamide) for four cycles followed by weekly Taxol (paclitaxel) for two weeks; stopped for neuropathy/ Month 5: right modified radical mastectomy; 10/10 positive lymph nodes/ Month 6-8: radiation therapy/ Month 11-12: Xeloda (capecitabine); stopped due to disease progression; two sites of disease recurrence, the skin and contralateral axillary lymph node (Stage IV), without any other distant metastasis; skin disease progression to the right arm / Month 13: patient presented to Fox Chase Cancer Center for treatment recommendations; PET/CT scan and brain MRI showed no metastatic disease other than the skin and contralateral axillary lymph node; CTC=1/ Month 14-17: Ixempra (ixabepilone) for five cycles; stopped on month 17 due to progressive disease in the skin; the erythema on her right chest wall continued to spread to the right upper quadrant of her abdomen and to the right mid-axillary line; CTCs= 0 (month 15); CTCs= 3* (month 16) (Stage IV); CTCs=1 (month 17) / Month 18: started gemcitabine and carboplatin; stopped after two cycles for progression skin disease and new mediatinal lymph nodes / Month 19: CTCs=2/ Month 20-21: Abraxane (paclitaxel) for two cycles, with lapatinib added with second cycle; stopped for progression of disease in the skin, left breast and left axilla; CTCs=9* (month 21) (Stage IV) / Month 21-24: started docetaxel, lapatinib and Tykerb (lapatinib); CTCs= 4 (month 22); CTCs= 0 (month 23), CTCs=1 (month 24); pleural effusions/ Month 24: metastatic inflammatory breast cancer confined only to the chest wall with minimal progression on PET/CT scan/ Month 25-26: began on IRB 09-017, a Phase I study of the anti-A5B1 integrin monoclonal antibody, PF-04605412; stopped due to disease progression; developed pleural effusions/ Month 26: started eribulin/ Month 30: patient started Cytoxan (cyclophosphamide), methotrexate and 5-fluorouracil (5-FU); CTCs= 6; CT chest revealed evidence of progressive disease on the chest wall and right axillary adenopathy; possibility of right chest wall posterolateral right lung involvement; no evidence of liver disease; the MRI of the brain was negative/ Month 31: patient died

*CTC values included in Table 1

**Patient J64403**

Month 0: IBC diagnosed in the left breast; stage IIIB; T4d N1 M0; triple negative/ Month 1-9: neoadjuvant chemotherapy with Adriamycin (doxorubicin ) and Cytoxan (cyclophosphamide) every two weeks for four cycles followed by weekly Taxol (paclitaxel) for twelve weeks; bilateral mastectomy with bilateral axillary node dissection; 4/24 positive lymph nodes; adjuvant radiation completed at month 9 / Month 9-21: disease free/ Month 22: skin changes that developed over the period of one day, consisting of small nodules in the area of her left chest wall previously irradiated; recurrent IBC diagnosis; no brain metastases; PET/CT scan showed left-sided pleural effusion and left supraclavicular node activity/ Month 24: patient presented to Fox Chase Cancer Center for treatment recommendations; local recurrence to chest wall with evidence of pleural effusion and a left supraclavicular node; CTCs=1* (month 24)/ Month 25-32: started Xeloda (capecitabine) and Ixempra (ixabepilone) for eight cycles; CTCs=0 (month 28); CTCs=0* (month 32)/ Month 33-37: PET scan showed bone metastases; started phase III study of Gemzar (gemcitabine), carboplatin, and a PARP inhibitor; CTCs=0 (month 37); stable disease although the patient has bone met; PET scan showed not metabolic activity in bone; the chest wall has responded/ Month 41-46: started the Iniparib (PARP inhibitor) and Gemzar; CTC=0 (month 43); CTCs= 9*(month 46)/ Month 48: patient died

*CTC values included in Table 1

**Patient D89802**

The patient had triple negative invasive ductal carcinoma (IDC) in the right breast seventeen months prior to IBC diagnosis; BRCA2 mutation positive; treated Taxotere (docetaxel), Adriamycin (doxorubicin) and cyclophosphamide for six cycles; bilateral mastectomy with right axillary lymph node dissection and saline implants for breast reconstruction; clinical trial randomized to receive cisplatin with PARP inhibitor for 4 cycles followed by an oral PARP inhibitor.

Month 0: IBC diagnosed in right reconstructed breast with positive lymph nodes in the right chest wall and right axilla and a lung lesion in the left upper lobule (Stage IV); triple negative/ Month 1: patient presented to Fox Chase Cancer Center for treatment recommendations; CTCs= 0/ Month 1-2: Abraxane (paclitaxel) for two cycles; progression in the right axillary, bilateral supraclavicular and hilar lymphadenopathy and in the skin of the right reconstructed breast/ Month 3-8: extensive skin disease on the right breast with ulcerations; start Ixempra (ixabepilone) and Xeloda (capecitabine); CTCs= 0 (month 5); CTCs=0 (month 6)/ Month 8: Ixempra and Xeloda stopped due to skin disease progression; CTCs=0/ Month 8-9: started carboplatin and Gemzar (gemcitabine); CTCs= 2*(month 9)/ Month 10-19: patient transferred to another institution; no data available (NDA)/ Month 19: patient died.

*CTC values included in Table 1

**Patient C65525**

Month 0: IBC diagnosed in left breast; Stage IIIB; T4d N1 M0; triple negative/ Month 1- 5: neoadjuvant Adriamycin (doxorubicin) and cyclophosphamide for four cycles given every four weeks followed by one cycle of Taxotere (docetaxel) with a significant bad reaction; patient was switched to Taxol (paclitaxel) every 3 weeks/ Month 7: left mastectomy with persistent extensive dermal lymphatic invasion with high-grade ductal involvement and 11/13 positive lymph nodes/ Month 8-9: radiation therapy / Month 11: patient presented to Fox Chase Cancer Center; no clinical evidence of disease; PET scan normal/ Month 14-15: developed local chest wall recurrence; skin rash that began in the right breast and extended to the chest wall of the previous surgery; biopsy proved to be recurrent IBC (Stage IV); CTCs=0* (month 15)/ Month 15-20: carboplatin and gemcitabine for 4 cycles; stopped due to skin disease progression; second chest wall recurrence; CTCs=0 (month 20)/ Month 20-21: capecitabine; stopped due to progressive disease in the chest wall/ Month 21-24: started Navelbine (vinorelbine); CTCs=0 (month 22); stopped due to skin disease progression; CTCs=0 (month 24)/ Month 24-26: started Ixempra (ixabepilone); stopped due to skin disease progression; the skin changes extended to the contralateral breast and the posterior left chest wall with diffuse redness; no ulceration; PET scan showed no bone or other site metastases/ Month 27: Doxil (doxorubicin) for one cycle while awaiting eligibility for Phase I clinical trial/ Month 27-28: skin disease progression with pruritus; CTCs=2 (month 27); started Avastin (bevacizumab); CTCs=8 (month 28); stopped due to skin disease progression; left inflammatory breast cancer with recurrent disease to the skin and right axillary lymph nodes (Stage IV) / Month 28-30: started clinical trial with ALK inhibitor (IRB 11-007); on month 29, improvement in the skin of the left chest wall, left back and right breast; CTCs= 2 (month 29); CT scan showed stable disease (month 29)/ Month 30: skin disease progression, with lesions covering a larger surface area; CTCs= 12* (month 30); left inflammatory breast cancer with recurrent disease to the skin and right axillary lymph nodes; stopped ALK inhibitor due to skin disease progression/ Month 30-33: started chemotherapy with Cytoxan (cyclophosphamide), methotrexate and 5-fluorouracil (5-FU); stopped on month 33 due to skin disease progression; CTCs= 4* (month 33)/ Month 34: PET scan showed bone metastases; spine metastases; minimal pleural fluid; CTCs=1 (month 34)/ Month 35: started compassionate treatment with gene therapy SGT-P53; stopped due to delirium; MRI revealed multiple brain metastases; whole brain radiation; metastatic disease to mediastinum, right axilla, and pleura as well as C7 in the setting of extensive chest wall disease / Month 38: Patient died.

*CTC values included in Table 1

**Patient B87480** (male)

Month 0: IBC diagnosed on his right breast; stage IV; triple negative/ Month 1-10: Taxotere (docetaxel), Adriamycin (doxorubicin) and cyclophosphamide one cycle; patient was then admitted to the hospital for neutropenic colitis; started dose-dense Adriamycin, Cytoxan (cyclophosphamide) for 3 cycles; remarkable skin response/ Month 11: weekly Taxol (paclitaxel) for seven weeks; stopped due to skin disease progression (new area of a rash on his right chest wall and his back); started Xeloda (capecitabine) 2 weeks on, 1 week off/ Month 13: patient presented to Fox Chase Cancer Center for treatment options; CTCs= 0* (month 13)/ Month 14-16: Ixempra(ixabepilone)/ Month 17: patient died.

*CTC values included in Table 1

**Patient R67904**

Month 0: IBC diagnosed; stage IV; T4d N3 M1; bone and liver metastases; CTCs= 6*/ Month 2- 9: Taxol (paclitaxel) for 12 cycles followed by 5-FU, cyclophosphamide and epirubicin for 6 cycles; CTCs= 0 (month 4);

CTCs= 0 (month 7); CTCs= 0 (month 8); CTCs= 0 (month 9)/ Month 10: PET scan showed multiple hepatic metastases and progressive disease in the same areas of liver and lymph nodes within her chest; CTCs= 0* (month 10)/ Month 11-12: started Ixempra (ixabepilone); stopped due to disease progression; residual disease, both in her left breast, liver and lymph nodes/ Month 13-15: started capecitabine; stopped due to disease progression specially liver metastases; CTCs=4 (month 15)/ Month 15-18: started gemcitabine and carboplatin; stopped due to disease progression; CTCs=3 (month 16); CTCs=2 (month 18)/ Month 18-20: started cyclophosphamide and methotrexate; stopped due to disease progression in liver and evidence of bone metastases; CTCs=15 (month 19); CTCs= 34* (month 20)/ Month 21: phase 1 LDK 378 (ALK inhibitor) one week; stopped due to diarrhea/ Month 22: patient died.

*CTC values included in Table 1

**Patient M76085**

Triple negative invasive ductal carcinoma (IDC) in the left breast, two years before IBC diagnosis; heavily treated with chemotherapy; bilateral mastectomy with expander placement.

Month 0: metastatic IBC in the left breast diagnosis; stage IV; metastases in skin and lymph nodes; triple negative/ Month 2-9: Ixempra (ixabepilone) followed by Xeloda (capecitabine)/ Month 10- 13: recurrent lesions in left breast; started carboplatin followed by radiation/ Month 13: PET CT scan showed spread of disease to the right axillary lymph node and left neck lymph node; started on Cytoxan (cyclophosphamide) for 2 weeks/ Month 14-18: Navelbine (vinorelbine); stopped due to disease progression on CT scan/ Month 19: gemcitabine for 4 cycles/ Month 20: patient developed pleural effusions and pulmonary abnormalities/ Month 21-27: Halaven (eribulin); stopped due to disease progression/ Month 27: patient presented to Fox Chase Cancer Center for treatment recommendations; CTCs= 0 (month 27)/ Month 27-29: started Abraxane (paclitaxel); stopped due to disease progression; CTCs=3* (month 29)/ Month 31: progressive disease in the skin and the lymph nodes; patient died.

*CTC values included in Table 1

**Patient M67752**

Breast cancer, ER+ PR+ Her2/neu-, in right breast two years before IBC diagnosis; stage III; lumpectomy and sentinel lymph node procedure followed by axillary resection; one positive lymph node. Dose-dense chemotherapy with Adriamycin and Cytoxan (cyclophosphamide), and radiation therapy, followed by endocrine therapy with Femara (letrozole) for a short time due that it was poorly tolerated.

Month 0: IBC in right breast, triple negative; contralateral (left) positive lymph nodes (Stage IV)/ Month 1-2: Taxotere (docetaxel) and Cytoxan (cyclophosphamide) for two doses; poorly tolerated and changed to Abraxane (paclitaxel) and Cytoxan with minimal response/ Month 3: patient presented to Fox Chase Cancer Center for treatment recommendations; CTCs=0* (month 3)/ Month 4-15: followed in another institution; bilateral mastectomy although it was not recommended at FCCC/ Month 8: lymph node recurrence on the right side; enrolled in a clinical trial with BSI PARP inhibitor with chemotherapy carboplatin and Gemzar (gemcitabine); initial response but after progressed; PET scan showed multiple active regions mainly on the right size (chest wall and lymph nodes); CTCs= 0* (month 15)/ Month 8-18: started Ixempra (ixabepilone); stopped due to disease progression; CTCs= 0 (month 18)/ Month 20-22: started trial on PARP 1 inhibitor; stopped due to disease progression/ Month 27-30: CMF followed by Doxil (doxorubicin); stopped to disease progression; PET scan showed progressive chest wall disease and some lymph node disease but no lung, no liver, no bone metastases; CTCs= 28* (month 30)/ Month 32: patient had extensive skin and lymph node disease but no liver, bone or lung disease; patient died.

*CTC values included in Table 1

**Patient E91111**

Month 0: IBC diagnosis in left breast; stage IIIB; triple negative/ Month 1-2: dose-dense Adriamycin (doxorubicin) and Cytoxan (cyclophosphamide) for four cycles / Month 3-5: started Taxol (paclitaxel) / Month 6: bilateral mastectomy which included a left radical mastectomy and left axillary lymph node dissection; 2/12 positive lymph nodes/ Month 7-8: adjuvant radiation therapy/ Month 12: metastases to the spine (Stage IV); radiation therapy/ Month 13-14: Ixempra (ixabepilone) and Xeloda (capecitabine); stopped due to disease progression/ Month 16-20: Zometa, gemcitabine and carboplatin; Zometa discontinued due to jaw pain / Month 21-24: continued gemcitabine and carboplatin/ Month 28-29: worsening bone metastases and started eribulin; stopped due to hospitalizations for chest pain, weakness, tingling in her hands and feet secondary to the eribulin/ Month 30: patient presented to Fox Chase Cancer Center for treatment recommendations; PET scan showed bone metastases and suspicious lesion in liver; CTCs=0* (month 30)/ Month 32-34: started Abraxane (paclitaxel); CTCs=8* (month 34); PET scan showed liver, bone and lung metastases; brain metastases was not determined since patient had expanders in place with metal that did not allow MRI / Month 34-36: started cisplatin/ Month 36: cisplatin two cycles completed; CTCs=0/ Month 37: started Taxotere (docetaxel)/ Month 39: bone, liver, lung metastases; patient died.

*CTC values included in Table 1

**Patient L95781**

Two years before IBC diagnosis, patient was diagnosed with right breast cancer (T1c N0 M0), triple negative; right mastectomy without reconstruction followed by 6 cycles of Taxotere (docetaxel), Adriamycin (doxorubicin) and cyclophosphamide.

Month 0: IBC diagnosis (Stage IV); triple negative; liver, bones and lymph nodes metastases/ Month 1-6: Taxotere (docetaxel) and carboplatin completed 8 cycles; stopped due to disease progression/ Month 7: patient presented to Fox Chase Cancer Center for treatment recommendation; CTCs=117* (month 7)/ Month 8: started Ixempra (ixabepilone) and Xeloda (capecitabine)/ Month 9: multiple liver metastases, small brain metastasis, bone and lymph node metastases; patient died.

*CTC values included in Table 1

**Patient L92225**

Month 0: IBC diagnosed in right breast (Stage IV; T4d N3 M1); triple negative; metastatic disease in in the axillary area but also the mediastinal and celiac region and there was development of right pleural fluid/ Month 1-3: chemotherapy with epirubicin and Cytoxan (cyclophosphamide) completed four cycles with good response/ Month 4-6: started Taxotere (docetaxel) and carboplatin for four doses; stopped due to disease progression/ Month 7-16: PET/CT showed a brachial plexopathy; Avastin (bevacizumab) and Ixempra (ixabepilone) with some response and persistent skin redness (inoperable disease); also received radiation and Xeloda (capecitabine) and Avastin; PET/CT showed progressive disease in the axillary area, mediastinal and celiac region, and development of right pleural fluid/ Month 14: patient presented to Fox Chase Cancer Center for treatment options; CTCs= 49 (month 14) (T4d N3 M1); patient progressed in the lymph nodes, pleural fluid, and skin/ Month 16: MRI showed no brain metastases; CAT scan of the chest revealed metastatic mediastinal lymph nodes, loculated right pleural effusion and skin thickening over the right breast and the chest wall and the abdominal region; no evidence of osseous metastatic disease/ Month 16-18: started Gemzar, Halaven, and Avastin; right pleural fluid with malignant cells; on month 18, the patient progressed clinically and radiographically; CTCs= 178* (month 18)/ Month 18-19: started Doxil and cyclophosphamide/ Month 20: patient died.

*CTC values included in Table 1

**Patient T89857**

Month 0: IBC diagnosed in left breast (Stage IIIB); triple negative/ Month 1-4: neoadjuvant dose-dense with Adriamycin and Cytoxan (cyclophosphamide) for four cycles; stopped due to disease progression (progressive erythema and purulence); started Taxol (paclitaxel)/ Month 4: patient presented to Fox Chase Cancer Center for treatment recommendations; PET scan showed extensive active disease in the skin of the breast and lymph nodes in the axilla; CTCs= 3* (month 4; Stage III)/ Month 4-7: Gemzar (gemcitabine) and carboplatin; followed by radiation therapy and Xeloda (capecitabine); on month 7, PET/CT scan showed a large contralateral lymph node suggestive of progressive disease; CTCs=2 (month 7; Stage IV); continued with Xeloda / Month 8: bilateral mastectomy; patient had residual disease and positive lymph nodes/ Month 9: Abraxane (paclitaxel) completed four cycles; followed by radiation therapy/ Month 11: CTCs=0* (month 11), T4d N2 M1 / Month 11-18: transferred care to another institution/ Month 19: metastatic disease in right breast and right axillary lymph nodes; patient died.

*CTC values included in Table 1

**Patient D66122**

Month 0: IBC in right breast; stage IIIB; T4d N1 M0; triple negative/ Month 1-4: neoadjuvant therapy with dose-dense Adriamycin (doxorubicin) and Cytoxan (cyclophosphamide) for four cycles followed by weekly Taxol (paclitaxel) for 8 weeks; stopped due to disease progression in breast and skin/ Month 5: patient presented to Fox Chase Cancer Center for treatment recommendations; PET/CT scan showed extensive residual disease in the right breast and lymph nodes/ Month 5-6: neoadjuvant carboplatin and Gemzar (gemcitabine) for four cycles; PET/CT showed minimal residual areas of residual disease in the breast/ Month 7: right breast modified radical mastectomy; patient had extensive residual disease and multiple lymph nodes 3/8 axillary lymph nodes with focal extracapsular extension/ Month 8-10: radiation therapy and Xeloda (capecitabine); followed by Xeloda as an adjuvant treatment; on month 10, the patient still had skin changes/ Month 11-15: continued with Xeloda; stopped due to erythema on the right chest wall along the incision line consistent with skin recurrence; PET CT showed evidence of disease recurrence in a right axillary lymph and left lymph nodes (Stage IV); MRI of the brain was negative and CT also did not show any evidence of pulmonary embolus; CTCs=21* (CTCs in clusters) (month 15)/ Month 15-17: Navelbine (vinorelbine) and cisplatin for two cycles; stopped due for neutropenic fever and pancytopenia as well as tinnitus secondary to cisplatin use and disease progression/ Month 18-22: Ixempra (ixabepilone) and Avastin (bevacizumab) for five cycles; CTCs=2 (month 21); stopped due to disease progression to chest wall and lymph nodes; CTCs= 5 (month 22)/ Month 22-24: eribulin and Avastin; stopped due to chest wall disease progression; CTCs= 23 (month 24)/ Month 25-26: skin disease continue to progress and increase activity in bone; started cyclophosphamide, methotrexate and 5-FU for 2 cycles; stopped due to disease progression; CTCs= 44 (month 25); CTCs= 140* (CTCs in clusters; month 26)/ Month 26: skin chest wall biopsy showed ErbB2 amplification by genomic analysis (Foundation Medicine); started Herceptin and Taxotere (docetaxel); completed one cycle; stopped due to skin disease progression; CTCs= 39* (month 27)/ Month 27-28: started carboplatin, Herceptin and lapatinib; stopped carboplatin and Tykerb (lapatinib) due to diarrhea/ Month 28-30: started Ixempra, Herceptin and Tykerb with metformin; CTCs= 109 (month 29); stopped after three weeks due to bacteremia; disease progression in chest wall and the other breast (left breast); CTCs=38 (month 30)/ Month 31: CTCs= 39* (CTCs in clusters); continued on Herceptin and lapatinib / Month 34: patient died.

*CTC values included in Table 1

**Patient K93878**

Month 0: IBC in left breast; stage III; triple negative/ Month 3-6: neoadjuvant chemotherapy with Taxotere (docetaxel), Adriamycin (doxorubicin) and cyclophosphamide for six cycles/ Month 7: bilateral mastectomy with immediate reconstruction followed by radiation to the left side/ Month 25: metastatic carcinoma to the right side and bone/ Month 26-32: Taxol (paclitaxel) along with Zometa / Month 32-37: started Xeloda (capecitabine); increased osseous metastasis especially in the ribs and hips; radiation therapy/ Month 37-40: Ixempra (ixabepilone) with Neulasta support, completed 6 cycles; stopped due to progression/ Month 41: started gemcitabine and Xgeva (denosumab); developed malignant pleural effusions/ Month 43-44: Adriamycin and Xgeva/ Month 44: patient came to Fox Chase Cancer Center for treatment recommendation; metastatic IBC with metastases to the bone, bladder, chest wall and malignant pleural effusion; CTCs= 127* (month 44); patient died

*CTC values included in Table 1

**ER-positive Her-2 negative IBC**

**Patient M71182**

The patient had ER+ (10%) PR+ (30%) Her-2/neu neg invasive ductal carcinoma (IDC) in the left breast one year before IBC diagnosis; patient underwent bilateral mastectomy with implants and reconstruction; 0/7 positive lymph nodes.

Month 0: IBC in left breast; stage IIIB; ER+ (10%) PR+ Her2-; skin nodules and chest wall recurrence on the surgical scar on the left side of the reconstructed breast / Month 1-3: Taxotere docetaxel), Adriamycin (doxorubicin) and Cytoxan (cyclophosphamide) for three cycles/ Month 4-13: additional disease within the surgical area; underwent DP flap, left breast removal of implant, standard radiation concomitant with carboplatin completed in month 8, and then received Navelbine (vinorelbine) for 12 weeks/ Month 14: started Tamoxifen; normal PET/CT scans until month 26/ Month 26- 29: lymph nodes in the internal mammary area were identified with a PET/CT, and suspicious area in the left lung; infection treated with fluconazole for three months/ Month 30: patient developed another recurrence in chest wall/ Month 31- 35: started on Abraxane (paclitaxel) and Avastin (bevacizumab) for six cycles/ Month 38: patient developed more nodules and rash; treated with antibiotics and Femara (letrozole); biopsy ER+ (28%) Her2/neu negative/ Month 40-43: photo-dynamic therapy/ Month 54: patient came to Fox Chase Cancer Center for treatments recommendations; stage IV; recurrent metastatic IBC; disease in the chest wall, the mediastinum, lymph nodes, liver and bone; no brain metastases; disease was progressing under current treatment with Femara; CTCs=0 (month 54)/ Month 54- 59: Xeloda (capecitabine) and Zometa; CTCs=0 (month 57); stopped Xeloda due to disease progression/ Month 60: increase in the ulceration of the chest wall; CTCs=3* (month 60) / Month 60-66: Ixempra (ixabepilone) and Xgeva (denosumab); CTCs=0 (month 63); CTCs= 7 (month 66); stopped due to disease progression in liver/ Month 66-69: started eribulin; CTCs= 11* (month 69); stopped due to disease progression; (significant erythema and skin thickening with nodularity, progression of disease in the skin, liver, bone, and lymph nodes)/ Month 70-71: Xgeva/ Month 71-75: started on Afinitor (mTor inhibitor) and Aromasin (exemestane; aromatase inhibitor); CTCs=48*(month 75); PET-CT showed significant progression of the disease in liver; stopped treatment and went to hospice care/ Month 76: extensive chest wall disease with liver and bone metastases; patient died.

*CTC values included in Table 2

**Patient D84055**

Month 0: IBC diagnosis in left breast, bone and lymph node metastases; stage IV; ER+ (80%) PR+ (90%) Her2/neu- / Month 1-6: neoadjuvant chemotherapy with Taxotere (docetaxel) for six cycles followed by Adriamycin (doxorubicin) and Cytoxan (cyclophosphamide) with minimal response; switched to Taxol (paclitaxel) and carboplatin for two cycles with partial remission/ Month 7-14: bilateral mastectomy; followed by radiation and Xeloda (capecitabine)/ Month 15: started Arimidex (anastrazole) for three years/ Month 51: PET/CT scan revealed multiple areas of distant recurrence in the right axilla, frontal bone, ribs, sacral bone and right acetabulum/ Month 52: patient came to Fox Chase Cancer Center for treatments recommendations; CTCs= 2*; metastatic inflammatory breast cancer to the bone and lymph nodes; brain MRI did not show any evidence of parenchymal disease/ Month 52-55: Faslodex (fulvestrant), Zometa and localized radiation to the humerus and pelvic bone; CTCs=0 (month 55)/ Month 61: continued on Faslodex; evidence of minimal progressive disease in the bone demonstrated by PET-CT; CTCs=0* (month 61)/ Month 62-69: started Aromasin (exemestane) and Afinitor (everolimus, mTOR inhibitor)/ Month 70-75: started Xeloda (capecitabine)/ Month 76-77: started PD-033299, a CDK4/6 inhibitor; developed pleural effusions on month 77/ Month 82: patient died.

*CTC values included in Table 2

**Patient M85099**

Month 0: IBC in right breast; stage IIIB; T4d N0 M0/ Month 2-5: neoadjuvant chemotherapy with Taxotere (docetaxel), Adriamycin (doxorubicin), and cyclophosphamide for six cycles/ Month 6: right breast mastectomy/ Month 7-9: adjuvant radiation therapy/ Month 9-18: adjuvant therapy with Tamoxifen (endocrine therapy)/ Month 14: developed metastasis in lung (Stage IV) /Month 18: patient came to Fox Chase Cancer Center for treatments recommendations; metastases in lungs, lymph nodes, bones and small left pleural effusion; no brain metastasis; CTCs= 1* (month 18)/ Month 18-25: started Ixempra (ixabepilone) and Xeloda (capecitabine); completed with excellent response by imagen and patient became asymptomatic by month 25; CTCs=1 (month 19); CTCs=0 (month 20); CTCs=0 (month 22); CTCs=0 (month 23)/ Month 26-28: discontinued Ixempra and continue Xeloda, and started with Arimidex (anastrazole) and Zometa/ Month 28: discontinued Xeloda, and continue with Arimidex as single agent and Zometa for bone metastases / Month 29: PET scan showed evidence of progressive disease; increase pleural fluid with metabolically active pleural disease; developed also metastatic disease in liver; multiple active lymph nodes in the upper abdomen possibly related to metastatic disease; CTCs=6* (month 29)/ Month 30-32: started second line endocrine therapy Afinitor (everolimus; mTOR inhibitor) and Aromasin (exemestane), along with Xgeva (denosumab; for bone metastases); CTCs=10 (month 31); stopped due to disease progression mainly in the liver; CTCs=11 (month 32)/ Month 32-36: changed from combination of endocrine therapy to chemotherapy with carboplatin and Gemzar (gemcitabine); stopped due to disease progression/ Month 36-41: started eribulin; stopped due to disease progression/ Month 41-44: started Doxil (doxorubicin); stopped due to disease progression/ Month 44-46: started Ixempra (ixabepilone); stopped due to disease progression/ Month 47: started clinical trial/ Month 50: metastatic disease in lungs, lymph nodes, bones and liver; patient died.

*CTC values included in Table 2

**Patient A89555**

Month 0: IBC diagnosis in right breast; stage III; ER+PR-Her2-/ Month 2-16: neoadjuvant chemotherapy with dose-dense Adriamycin (doxorubicin) and Cytoxan (cyclophosphamide) for four cycles; after mastectomy that showed extensive residual disease; 22/23 positive lymph nodes; adjuvant chemotherapy with dose-dense Taxol (paclitaxel), and endocrine therapy with Tamoxifen / Month 18-19: radiation therapy/ Month 19: fibroadenoma in left breast / Month 32: patient developed bone metastases in femur and sacrum; radiation therapy and surgery on right hip/ Month 32- 42: endocrine therapy with Tamoxifen, after changed to Arimidex (anastrazole) and Lupron (leuprorelin); and later changed to Aromasin(exemestane)/ Month 43-47: started Xeloda (capecitabine); stopped due to PET/CT scan showed progressive disease in the lymph node in the abdomen and the bones/ Month 46-47: started eribulin/ Month 47: patient came to Fox Chase Cancer Center for treatments recommendations; metastatic IBC (Stage IV) with bone and lymph node metastases; CTCs=1* (month 47) / Month 47-50: continued chemotherapy erilubin; completed it with maximum response; CTCs=0 (month 49); CTCs=0 (month 50)/ Month 50-56: started endocrine therapy combination with Aromasin and Afinitor (everolimus; mTOR inhibitor), and Xgeva (denosumab, for bone metastases); CTCs=0 (month 53); CTCs=0 (month 54); CTCs=0* (month 55)/ Month 57- 100: no data available (NDA)/ Month 101: patient died.

*CTC values included in Table 2

**Patient M66830**

Month 0: IBC in left breast diagnosis; stage IIIC; T4d N3 M0; ER+ (70%) PR- (0%) Her2/neu -; patient came to Fox Chase Cancer Center for treatment; CTCs= 345*/ Month 1-5: neoadjuvant chemotherapy with Adriamycin (doxorubicin) and cyclophosphamide for four cycles followed by Taxol (paclitaxel) for four cycles/ Month 6: left modified radical mastectomy and axillary lymph node dissection; 7/7 positive lymph nodes/ Month 7-9: adjuvant radiation therapy/ Month 9-15: started adjuvant endocrine therapy with Tamoxifen; in month 15, no evidence of distal metastases or brain metastases/ Month 16: developed metastases in bone (vertebral area); CTCs=0 (month 16); started Arimidex (anastrazole; endocrine therapy) and Zometa/ Month 19: metastatic lobular carcinoma of the breast, bones, in the bilateral ovaries and fallopian tubes; progressive disease in bones; CTCs=4* (month 19); started Faslodex (fulvestrant) and Xgeva (denosumab, for bone disease)/ Month 22: PET scan showed increased metabolic activity in bones and suspicious lesion in the liver; an MRI of the cervical and thoracic spine was performed which showed enhancement in the cervical spine concerning for leptomeningeal disease; a brain MRI showed no evidence of metastatic disease although there was again enhancement worrisome for leptomeningeal disease; CTCs= 66 (month 22); changed to chemotherapy with Xeloda (capecitabine), and continue with Xgeva / Month 23-26: leptomeningeal and bone disease; started Xeloda and intrathecal methotrexate; CTCs=189* (month 26) / Month 27-31: no data available (NDA)/ Month 32: PET scan showed progression of the disease with new sites of metastases in the bone, multiple liver lesions and some peritoneal uptake as well; also new lung nodules; started erilubin / Month 33: disease progressing; brain metastases; whole brain radiation/ Month 37: metastases in bones and bilateral ovaries, brain metastases; patient died.

*CTC values included in Table 2

**ER-negative Her2 positive IBC**

**Patient N88166**

Month 0: IBC on right breast; stage IIIB; T4d N1 M0; ER- PR- Her2/neu + (3+ by IHC)/ Month 1: PET/CT scan of the chest, abdomen, and pelvis and bone scan showed no evidence of metastatic disease/ Month 1-2: patient declined treatment and went for alternative therapies; on month 2, disease progressed with new skin changes on the contralateral (left) breast and axilla/ Month 3-7: neoadjuvant therapy with Taxotere (docetaxel), carboplatin and Herceptin for six cycles; near complete response/ Month 8-10: started weekly Herceptin but stopped due to disease progression (recurrence of erythema and nodularity in the left breast)/ Month 11-15: started carboplatin, Abraxane (paclitaxel), and Herceptin with 5-fluorouracil (5-FU) added at the 4^th^ cycle because there was no significant response of the skin disease; stopped due to disease progression in the skin with increased erythema and nodularity over both breasts/ Month 16-18: started Herceptin, Navelbine (vinorelbine), and Tykerb (lapatinib); stopped for to clinical disease progression/ Month 18: patient came for the first time to Fox Chase Cancer Center for treatment recommendations; brain metastases were found (three small lesions in the brain); CTCs= 1* (month 18)/ Month 18-21: started epirubicin and Cytoxan (cyclophosphamide); stopped due to disease progression in the skin of the chest wall and abdomen, and minimal increase in one brain lesion; CTCs=7* (month 21)/ Month 22: patient had acute delirium and was hospitalized/ Month 23: disease progression in the skin of chest wall and abdomen with brain metastases; patient died.

*CTC values included in Table 2

**Patient S71769**

Month 0: IBC in left breast diagnosis; stage IIIB; T4d N1 M0; ER- PR- Her2/neu + (3+ by FISH)/ Month 2: neoadjuvant chemotherapy with Adriamycin (doxorubicin) and cyclophosphamide for one cycle. Patient transferred care to Fox Chase Cancer Center/ Month 3-8: started neoadjuvant chemotherapy with 5- fluorouracil (5-FU), epirubicin, and cyclophosphamide for three cycles followed by 12 weeks of paclitaxel; also Herceptin started on month 3 for one year; complete clinical response/ Month 8-14: left breast mastectomy and axillary dissection; with complete pathological response, followed by radiation therapy and continued Herceptin (for one year; completed in month 14)/ Month 15: no evidence of recurrent disease/ Month 17: MRI showed metastasis in brain (single lesion)/ Month 18: brain metastasis treated with surgery and stereostactic radiation; CTCs=0* (month 18) / Month 19-27: started Tykerb (lapatinib) and Xeloda (capecitabine) for metastatic IBC/ Month 27: no evidence of recurrent disease/ Month 28-58; no data available (NDA)/ Month 59: patient died

*CTC values included in Table 2

**Patient I77438:**

Month 0: IBC diagnosis in the right breast with metastases in bone and liver; stage IV; T4d N1 M1; ER- PR+ (20%) Her2/neu + (3+ by IHC)/ Month 1-5: Taxotere (docetaxel), carboplatin and Herceptin for six cycles without receiving Zometa (for bone metastases); continued with Herceptin/ Month 7-9: continue with Herceptin and started Tamoxifen/ Month 9: patient developed large lesion in brain with edema (brain metastasis was ER/PR- neg Her2/neu- positive)/ Month 10: brain surgery/ Month 11: patient came to Fox Chase Cancer Center for evaluation and treatment recommendation; CTCs=0* (month 11)/ Month 11-18: stopped the treatment with Herceptin and Tamoxifen and started on Xeloda (capecitabine) and lapatinib; stopped due to disease progression in the lymph nodes and brain; gamma knife treatment of two additional brain metastases on month 14; CTCs=0 (month 14)/ Month 18-21: started Xeloda, lapatinib and Herceptin; stopped due to disease progression in the right breast/ Month 21-26: started Herceptin, lapatinib, oral metronomic cyclophosphamide and methotrexate; stopped due to skin disease progression/ Month 27-30: started erlotinib (epidermal growth factor receptor inhibitor), Herceptin, oral metronomic cyclophosphamide and methotrexate; stable disease on month 30/ Month 31-38: no data available (NDA)/ Month 39: metastatic disease to the bone, liver and brain; patient died (see Ref. 17: Ali et al, 2014: Clin Breast Cancer 14: 14-16).

*CTC values included in Table 2

**ER-positive Her2-positive IBC**

**Patient L88046**

Month 0: IBC diagnosis in right breast; patient was breastfeeding at the time of diagnosis; breast tissue biopsy was ER+ PR- Her2/ neu + (3+ by IHC); stage IV; T4d N1 M1; metastatic carcinoma in the contralateral (left) axillary lymph node that was ER/PR negative HER-2 + / Month 1-6: neoadjuvant therapy with Adriamycin (doxorubicin) and cyclophosphamide followed by Taxol (paclitaxel) with Herceptin; stopped due to disease progression/ Month 7-9: Xeloda (capecitabine) and Tykerb (lapatinib) for three months with clinical response/ Month 10-11: bilateral mastectomy; 0/10 positive lymph nodes in the right side and 0/15 positive lymph nodes in the left side; radiation therapy / Month 12-18: Xeloda and Tykerb for six month / Month 18- 20: stopped Xeloda; continued with Tykerb, and added Tamoxifen (because of the original ER positivity on the first biopsy); stopped due to skin disease progression and bilateral pleural effusions/ Month 21-27: started Herceptin, Ixempra (ixabepilone) and Tykerb; Ixempra was discontinued on month 27 after 8 cycles due to toxicity; pleural effusions started to re-accumulate/ Month 27-31: started Xeloda and Tykerb; CT scan on chest and abdomen demonstrated increased bilateral pleural fluid with near complete collapse of the right lower lung and ascites and an ovarian abnormality suggestive of peritoneal disease and ovarian metastasis/ Month 31: patient came to Fox Chase Cancer Center for treatment recommendations; currently metastatic disease to the ovaries with peritoneal disease and brain metastases; pleural fluids and ascites; stopped Xeloda and Tykerb due to disease progression; CTCs=1 (month 31); whole brain radiation followed by stereostatic radiotherapy/ Month 31-35: started Taxotere (docetaxel), carboplatin and Herceptin; CTCs=0 (month 33); pleural effusion positive for tumor cells that were ER- PR- Her2+; achieved maximum response on month 35/ Month 36-38: continued carboplatin and Herceptin; stopped due to disease progression in the ovary, bilateral pleural fluid, mediastinal disease, most likely lymph nodes or soft tissue, and possibility of lymphangitic spread within the lung/ Month 38-40: Abraxane (paclitaxel), Herceptin and Tykerb for five weeks; stopped due to disease progression; pleural effusions ER- PR- Her2+ (2+ by IHC); CTCs=5* (month 39)/ Month 41-42: discontinued Tyker, continue paclitaxel and Herceptin, and started Gemzar (gemcitabine)/ Month 43: metastatic disease to the pleura, ovaries, abdomen, lymphangitic spread and small osseous traumatic brain metastasis; patient died.

*CTC values included in Table 2

**Patient J70105**

Fourteen years before IBC, the patient had ductal carcinoma in situ (DCIS) in her left breast treated with lumpectomy followed by radiation therapy.

Month 0: IBC in left breast diagnosis; stage III; ER+ (90%) PR+ Her2/neu +/ Month 1-5: started neoadjuvant therapy with Taxotere (docetaxel), carboplatin and Herceptin for six cycles with significant clinical response/ Month 6: mastectomy of the left breast with left axillary lymph node dissection and flap reconstruction; 0/15 positive lymph nodes; started Arimidex (anastrazole) and Herceptin/ Month 11: area of redness on the chest wall of the reconstructed breast area; progressive disease in skin and chest and dermal lymphatics; patient came to Fox Chase Cancer Center for treatment recommendation; MRI showed no brain metastasis /Month 12-22: started Tykerb (lapatinib) and Aromasin (exemestane) followed by radiation therapy; patient had a skin recurrence (ER+ PR- Her2/neu +); CTCs=0* (month 22)/ Month 22-28: continued Tykerb (lapatinib), Aromasin and Herceptin was added; CTCs=0 (month 26); stopped due to disease progression / Month 28-31: started Ixempra (ixabepilone) every three weeks in combination with Herceptin and daily Tykerb (lapanitib); good response; CTCs=0 (month 31)/ Month 32-79: non data available (NDA)/ Month 80: patient is alive.

*CTC values included in Table 2

**Patient K76386**

Month 0: IBC diagnostic in right breast; ER+ (10%) PR-, Her2 + (3+); stage IIIC; T4d N3 M0/ Month 2-6: neoadjuvant chemotherapy with Taxotere (docetaxel), carboplatin and Herceptin for six cycles/ Month 7: right mastectomy and axillary lymph node dissection; pathology showed residual invasive ductal carcinoma with micropapillary features, grade 3, multifocal; 11/30 positive lymph nodes with extranodal extension/ Month 8-15: started adjuvant therapy with Herceptin and Arimidex (anastrazole) / Month 9: patient came to Fox Chase Cancer Center for treatment recommendation; right breast upper core needle biopsy ER + (10% weak), PR-(0%) Her2/neu + (3+ by IHC); right local lymph node was Her2 negative by FISH (1.3)/ Month 9-12: radiation therapy; on month 12, no evidence of residual or recurrent disease/ Month 15: disease progression on Herceptin and Arimidex; extensive redness and erythema; open areas in her skin in the right chest wall; developed pleural effusion with malignant cells/ Month 15-17: started 5-FU, epirubicin and cyclophosphamide (FEC) combined with Tykerb (lapatinib); CTCs=111 (month 15); CTCs=32* (month 17)/ Month 17-19: stopped Tykerb due to toxicity, and continued FEC and reintroduced Herceptin; on month 18, clinically evidence of response; CTCs= 9 (month 18); CTCs= 24 (month 19); on month 19, disease progression; PET scan showed progressive disease in skin and some bone lesions; invasion to the liver parenchyma cannot be completely excluded; no brain metastasis/ Month 20: started Abraxane (paclitaxel)/ Month 21: extensive skin disease, pleural effusion with malignant cells, bone metastases, and possible liver metastases; patient died.

*CTC values included in Table 2

**Patient B62630**

Month 0: IBC in left breast; ER+ PR+, Her2 +; stage III/ Month 6-11: neoadjuvant therapy with Adriamycin (doxorubicin) and cyclophosphamide every two week for two cycles with no response; TAC for 4 cycles; stopped for the develop of contralateral disease/ Month 10-11: Xeloda (capecitabine); radiation/ Month 12: bilateral mastectomy; 8/13 positive lymph nodes/ Month 14-21: started Herceptin, Tamoxifen and Zometa/ Month 22: disease recurrence in skin/ Month 23-29: Tykerb (lapatinib) and Xeloda; stopped due to disease progression in the skin/ Month 32-41: Zoladex (goserelin) and Femara (letrozole); patient did not receive chemotherapy/ Month 42: disease progression with new pleural effusions/ Month 42-44: Zoladex and Faslodex (fulvestrant)/ Month 44-46: Zoladex; progression in the skin, pleural effusion and mediastinal lymph nodes/ Month 46-49: patient discontinued all treatment; continued to have progression in the skin and pleural effusions/ Month 49: patient presented to Fox Chase Cancer Center for treatment recommendations; metastatic IBC to the skin and pleura (Stage IV); PET/CT scan showed hypermetabolic mediastinal and bilateral lymph nodes, evidence of multiple areas of disease, pleural thickening in the left side, and also chest wall disease; CTCs= 29*(month 49); tumor was ER+ PR- Her2- / Month 50: Ixempra (ixabepilone) for two cycles; stopped due to allergic reaction after second cycle and progression of the disease in the skin and lungs with lymphangitic spread/ Month 51- 53: started carboplatin, Herceptin and Tykerb (lapatinib); CTCs= 43* (month 51); CTCs=19 (month 52); CTCs= 36 (CTCs in clusters; month 53); on month 53, stopped due to disease progression in the skin/ Month 54-55: metastases in right lung; radiation therapy/ Month 56: CTCs= 28 (month 56); metastatic left inflammatory breast cancer with disease in the right breast, skin, lymph nodes and pleura; started with Doxil (doxorubicin); patient died.

*CTC values included in Table 2
